# Seasonality of underweight among infants 1–11 months old in Niger: an exploratory analysis of data from a cluster-randomised trial

**DOI:** 10.1136/bmjgh-2024-017643

**Published:** 2025-03-28

**Authors:** Brittany Peterson, Ahmed Mamane Arzika, Ramatou Maliki, Amza Abdou, Bawa Aichatou, Ismael Sara, Diallo Beidi, Nasser Galo, Nasser Harouna, Alio Karamba Mankara, Sani Mahamadou, Moustapha Abarchi, Almou Ibrahim, Elodie Lebas, Jeremy David Keenan, Catherine E Oldenburg, Travis C Porco, Benjamin Arnold, Thomas M Lietman, Kieran S O’Brien

**Affiliations:** 1 Francis I. Proctor Foundation, University of California San Francisco, San Francisco, California, USA; 2 Centre de Recherche et Interventions en Sante Publique, Niamey, Niger; 3 Carter Center, Niamey, Niger; 4 Programme Nationale de Santé Oculaire, Niamey, Niger; 5 Centre de Recherche et Interventions en Santé Publique, Birni N’Gaoure, Niger

**Keywords:** Global Health, Cluster randomized trial, Child health

## Abstract

**Introduction:**

Malnutrition is a risk factor for child mortality, with around 45% of deaths in children under 5 globally linked to malnutrition. Seasonality of malnutrition has important implications for the timing of child health programme activities, but evidence is mixed on the nature of such patterns. Moreover, the bulk of the existing evidence is focused on wasting and stunting in children 6–59 months, despite increasing evidence that younger children also face a high risk, and that underweight alone is an important predictor of mortality.

**Methods:**

This study used data from the cluster-randomised AVENIR trial which compared the effect of biannual distribution of azithromycin vs placebo on mortality in children 1–59 months old in Niger. AVENIR included a biannual census conducted on a rolling basis over 2 years. A subset of 133 781 infants aged 1–11 months from 2904 communities were included in this study, and weight-for-age z-score (WAZ) was calculated at each census. The exposure for this analysis is the day of the year weight was captured. Harmonic regression was used to determine primary and secondary peaks and nadirs of WAZ over time.

**Results:**

Overall, the primary peak of WAZ occurred in late February and the primary nadir occurred in mid-May, aligning with a seasonal temperature increase before the rainy season. A secondary peak in August and a secondary nadir in November were also seen, aligning with the postrainy season.

**Conclusion:**

The seasonality of WAZ of infants 1–11 months in Niger may have implications for the timing of programmes aiming to decrease malnutrition.

**WHAT IS ALREADY KNOWN ON THIS TOPIC**

- There is conflicting evidence that growth patterns in children in Sub-Saharan African settings follow a seasonal pattern. More research is needed to understand timing and seasonality for purposes of programme implementation targeting malnutrition.

**WHAT THIS STUDY ADDS**

- Among infants in rural areas of Niger, the primary peak of WAZ occurred in late February and the primary nadir occurred in mid-May, aligning with a seasonal temperature increase before the rainy season. A secondary peak in August and a secondary nadir in November were also seen, aligning with the post-rainy season.

**HOW THIS STUDY MIGHT AFFECT RESEARCH, PRACTICE OR POLICY**

- The seasonality of WAZ of infants 1–11 months in Niger highlights critical periods of increased vulnerability which can inform optimal timing of malnutrition and mortality prevention and intervention programmes. This study provides quantitative evidence which can help optimise resource allocation and anticipate high-risk periods of undernutrition.

## Introduction

 The United Nations Sustainable Development Goal 2 for 2030 aims to eliminate all forms of hunger and malnutrition.^1^ Sub-Saharan Africa is currently off-track to reach this goal by 2030.^2^ Malnutrition in children is a risk factor for mortality, and around 45% of deaths of children under 5 globally are associated with malnutrition.^3^ Despite widespread emphasis on wasting and stunting, underweight, as measured by weight-for-age z-score (WAZ) is a particularly strong predictor of mortality.^4^,^6^ Niger has one of the highest prevalences of children with underweight status in Sub-Saharan Africa at 36.4%.^7^ Underweight status has been shown to be related to mortality risk through diarrhoeal disease and respiratory infections, which are known to follow seasonal patterns in the Sahel.^3^

There is conflicting evidence that growth patterns in children in African settings follow a seasonal pattern. Multiple studies have identified the rainy season as the time of year with the worst malnutrition outcomes.^8 9^ Contradicting these findings, other studies have determined that wasting and/or stunting were most common during the dry season.^10^,^12^ Possible explanations for the mixed findings include the use of crude seasonal categorisations, like rainy versus dry season and preharvest versus postharvest, as well as the aggregation of data by month. Such aggregation of seasonal data can lead to a loss of precision and has the potential to result in inaccurate or biased conclusions.^8 13^ Multiple factors during one season or across seasons can lead to fluctuations in nutritional status such as rainfall, temperature, food insecurity, and time of harvests, which also vary spatially within a season.^8^

Most studies on seasonality of malnutrition have focused on children 6–59 months of age, aligning with prior programme priorities. However, more recent guidelines have emphasised the need to include infants under 12 months of age as well.^14^ The AVENIR adaptive cluster-randomised trial collected weight and age data on infants 1–11 months old as part of a rolling census across all seasons over 2 years.^15^ This exploratory analysis aims to leverage that data to assess the seasonality and spatial distribution of WAZ in Niger. We hypothesise that the WAZ of children 1–11 months in this trial will vary spatially and by season.

## Methods

### Study design and setting

This study used data from the adaptive cluster-randomised AVENIR trial which compared the effect of biannual distribution of azithromycin versus placebo on mortality in children 1–59 months old.^15 16^ AVENIR included 3000 communities within the regions of Dosso and Tahoua with 382 586 children 1–59 months old. Communities were randomised to one of three arms: azithromycin for children 1–59 months, azithromycin for children 1–11 months and placebo for children 1–59 months. The trial conducted a census of each household in the study area every 6 months between November 2020 and July 2023. Census data collection included demographics, vital status and dosing details for children 1–59 months of age. The dose was determined using weight for infants 1–11 months old. For children over 11 months, dose was determined using height as an approximation for weight for children able to stand or weight for children unable to stand.^17^ The census was done on a rolling basis so data were captured throughout the year.

### Patient and public involvement

Neither patients nor the public were involved in the design of this study. However, the Niger Ministry of Health contributed to the study’s design. Additionally, the Niger Ministry of Health, health post leaders, community health workers and community leaders were engaged in the recruitment, implementation and dissemination phases of the study.

The Comité National Éthique pour la Recherche en Santé in Niger (# 041/2020/CNERS) and the Institutional Review Board (IRB) (#19–28387) at the University of California, San Francisco provided ethical approval. A Data and Safety Monitoring Committee oversaw participant safety and study progress through quarterly reports and annual meetings. Verbal informed consent was obtained from community leaders before study activities began. Heads of household provided verbal consent before census activities, as well as caregivers of eligible children before treatment administration. Written consent for infants aged 30–42 days was obtained from caregivers before treatment due to the risk of macrolide-associated infantile hypertrophic pyloric stenosis.^18^ The trial was registered at clinicaltrials.gov (NCT04224987) on 13 January 2020.

### Study participants

For this study, only infants aged 1–11 months old were included since older children did not have weight recorded to determine dose. Infants were included in this analysis if they had at least one census visit with valid weight and age recorded. Infants with WAZ less than −6 or greater than 5 were excluded based on WHO recommendations.^19^

### Data collection and variables

The outcome for this analysis is average community-level WAZ at the time of the census. WAZ was determined using the ‘anthro’ package in R.^20^ This package calculates the WAZ according to WHO guidelines and uses the weight, age and sex of the participant to calculate z-scores compared with a global reference population.^21^ Weight was taken by trained study personnel using a hanging scale and recorded to the nearest 0.1 kg (ADE M111600, GmbH & Co., Hamburg, Germany). Weight was only taken if the child was eligible for treatment and present while treatment was being delivered. Children were eligible for treatment if they were 1–59 months old at the time of treatment, weighed at least 3 kg and had no known allergy to macrolide antimicrobials. Age was recorded at the time of the census by using date of birth from the child’s health card if available or asking the child’s caregiver/guardian for the child’s age in months. Sex was recorded at each census as well. Finally, mean WAZ by community was computed.

The exposure for this analysis is the day of year weight was captured, pooled across years. Secondary analyses examined day of year separately for the years of 2021 and 2022 as well as for ages 1–5 months and 6–11 months. A sensitivity analysis looked at the day of the year pooled to four seasons: dry, prerainy, rainy and postrainy, using countrywide rainfall data to identify the 3 months with the highest rainfall as the rainy season. Seasons were then categorised into 3-month intervals based on rainy season. Rainfall and temperature data were sourced from the Climate Research Unit gridded Time Series monthly climate datasets.^22^ All census data were captured using electronic data collection through the CommCare mobile application (Dimagi, Cambridge, MA USA).

### Sample size and statistical analysis

AVENIR was designed to have 80% power to detect a 10% difference in all-cause mortality at an alpha of 0.05 comparing the 1–59-month azithromycin and placebo arms. AVENIR data included 2904 communities and 136 673 total infants with at least one census data collection while aged 1–11 months, and our study population was fixed to this number.

Analyses were conducted in R V.4.2.2 (R Foundation for Statistical Computing, Vienna, Austria). Descriptive characteristics of participants were summarised at the community level by season reporting mean and SD for age, proportion female, and mean and SD for WAZ. To determine seasonal trends and identify primary and secondary peaks and nadirs of WAZ, harmonic regression at the community level was used. Models with 1, 2 and 3 harmonics were compared using Akaike Information Criterion (AIC), and the model with the lowest AIC was selected. This method has been used in previous seasonality analyses.^1223^,^26^ The model used in this analysis is as follows:

$$\begin{array}{l} Y_{i}=\beta_{0}+\beta_{1}day_{i}+\beta_{2}\sin(2\pi \times \frac{day_{i}}{365})+\beta_{3}\cos(2\pi \times \frac{day_{i}}{365})\\ \;+\beta_{4}\sin(4\pi \times \frac{day_{i}}{365})+\beta_{5}\cos(4\pi \times \frac{day_{i}}{365})+\epsilon_{i} \end{array}$$

The primary and secondary peaks and nadirs were identified by inflection points, and the date of each is reported in a table. A graph illustrates the predicted values of WAZ by day of the year. The coefficient for each term, as well as 95% CIs and *P* values are reported in supplemental material.

Secondary stratified analyses include subgroups defined by year, age group and treatment received. The subgroup by year only includes the years 2021 and 2022 for which data was fully collected throughout the year. Age subgroups include 1–5 months and 6–11 months old. Treatment received is classified as either azithromycin for communities belonging to the azithromycin 1–59 months arm or azithromycin 1–11 months arm and placebo for communities belonging to the placebo 1–59 months arm. As a supplemental analysis, the mean difference in WAZ by categorical season was determined using a linear regression model with data pooled at the village level and dry season as the reference. Another supplemental analysis looking at mean difference in prevalence of underweight as defined by WAZ −2 was determined in the same way. Mean difference, *P* values, and 95% CIs are reported.

To illustrate the spatial distribution of WAZ, a simple kriging approach was used. The kriging model was fit with a spatial regression model with spatial covariance using the Matern covariance function with smoothness *v* and scale *p* parameters. The ‘spam’ package in R was used.^27^ Using the geostatistical model fit, predictions were made over a 50-kilometre radius around the study communities to estimate the average WAZ during each season.

## Results

**Figure 1:**
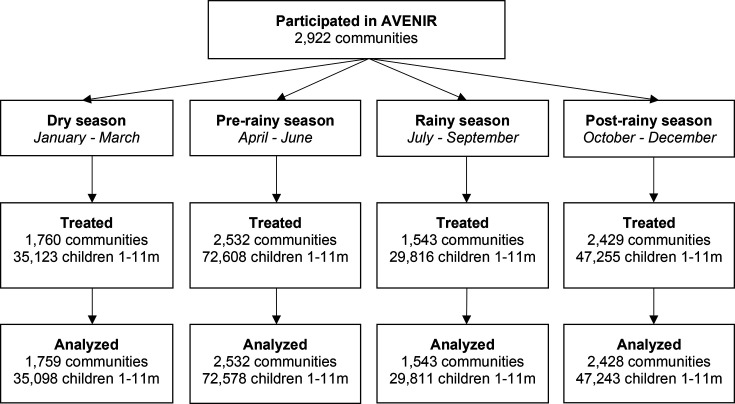
Participant flow.

Overall, 136 673 infants 1–11 months participated in the AVENIR trial (figure 1). Of these infants, exclusions included 2657 infants who did not have weight recorded at any timepoint due to ineligibility for treatment or absence from the community during treatment distribution and 235 infants with outlying WAZ. A total of 133 781 infants and 185 965 observations contributed to analyses. Community-level baseline characteristics are displayed in table 1. Mean age of infants was slightly higher in the dry and prerainy seasons, 5.5 months (SD=1.5) and 5.4 months (SD=1.2), respectively, compared with the postrainy and rainy seasons, 5.1 months (SD=1.4) and 5.3 months (SD=1.3), respectively. The proportion of female participants remained constant across all four seasons. Average WAZ was lowest in the postrainy season (−1.1, SD=0.9) and prerainy season (−1.1, SD=0.7), compared with the dry and rainy seasons (−1.0, SD=0.8 and −1.0, SD=0.8, respectively).

**Table 1:** Baseline characteristics at the community level of participants from first entry into the census

Characteristic	Prerainyn=2496	Rainyn=1470	Postrainyn=2263	Dryn=1559	Overalln=7788
Children per cluster(mean, SD)	24.4 (32.0)	15.6 (13.9)	11.5 (10.9)	15.4 (17.9)	17.2 (18.2)
Age in months(mean, SD)	5.4, 1.2	5.3, 1.3	5.1, 1.4	5.5, 1.5	5.3, 1.3
Percent female(mean, SD)	50.1%, 16.9%	49.9%, 20.2%	49.7%, 23.6%	49.3%, 23.1%	49.7%, 20.9%
WAZ(mean, SD)	−1.1, 0.7	−1.0, 0.8	−1.1, 0.9	−1.0, 0.8	−1.1, 0.8

WAZweight-for-age z-score

The results of the harmonic regression model suggest a significant 6-month seasonal cycle in WAZ (sin4π of day=0.19) (95% CI 0.13 to 0.25, *p* value<0.001) with two peaks per year (table 2).

**Table 2:** Harmonic regression coefficients, 95% CIs and *P* values for the overall seasonal trends of WAZ

Coefficient	Estimate(95%CI)	P value
Day	0.00 (0.00 to 0.00)	0.0029
sin2π of day	0.09 (0.01 to 0.17)	0.0345
cos2π of day	−0.02 (−0.07 to 0.02)	0.3014
sin4π of day	0.19 (0.13 to 0.25)	<0.001
cos4π of day	−0.02 (−0.07 to 0.02)	0.3306
Constant	−1.26 (−1.38 to −1.15)	< 0.001

WAZweight-for-age z-score

**Figure 2:**
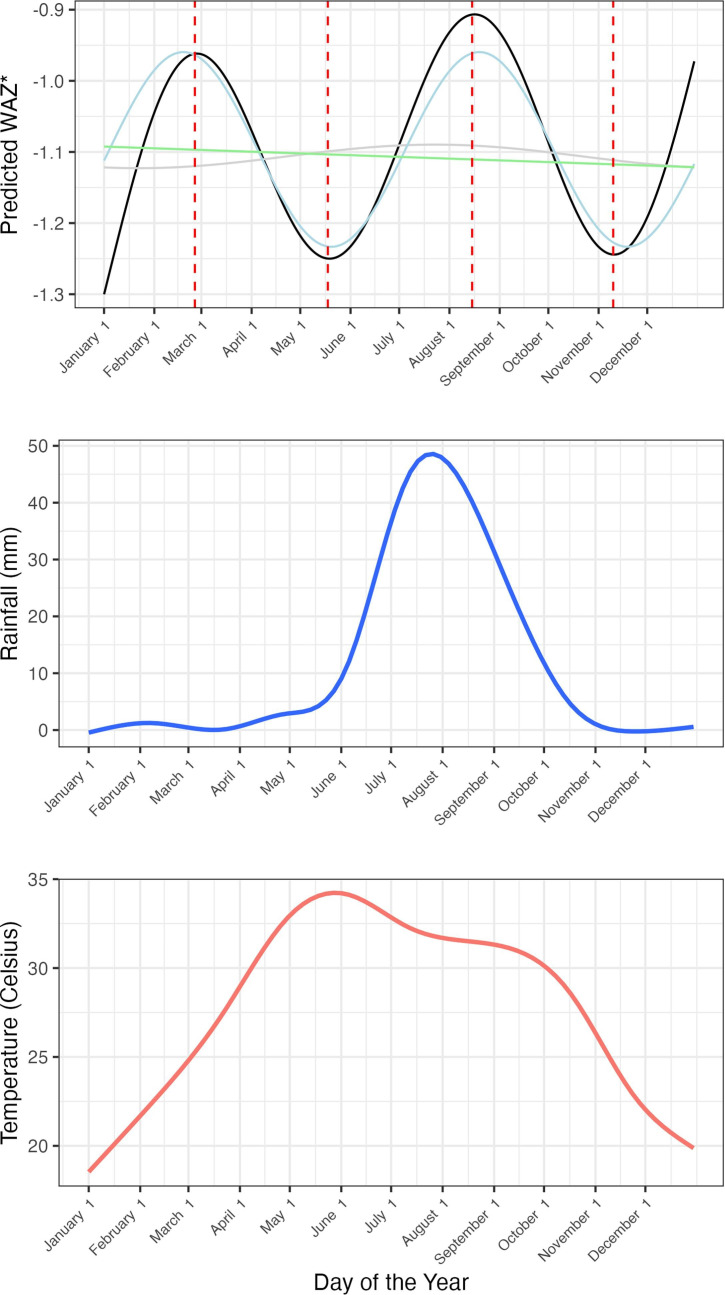
Predicted daily values of WAZ from the harmonic regression model with average monthly temperature and rainfall.*Graph depicts the annual curve alone in grey, the biannual curve alone in blue, and the time trend alone in green.

The harmonic regression model indicated that the primary peak of WAZ occurred in late February with a secondary peak in mid-August (table 3, figure 2). The primary nadir of WAZ occurred in mid-May and the secondary nadir in November.

**Table 3:** Predicted timing of primary and secondary peaks and nadirs for weight-for-age z-score

Group	Primary peak	Primary nadir	Secondary peak	Secondary nadir
Overall	February 26	May 18	August 15	November 10
Year				
2021	February 04	May 09	August 06	November 15
2022	March 12	June 02	August 30	November 18
Age Group				
1–5 m	February 17	May 17	August 15	November 11
6–11 m	February 22	May 26	August 15	October 14
Treatment				
Azithromycin	February 26	May 18	August 14	November 5
Placebo	February 19	May 10	August 14	November 25

**Figure 3:**
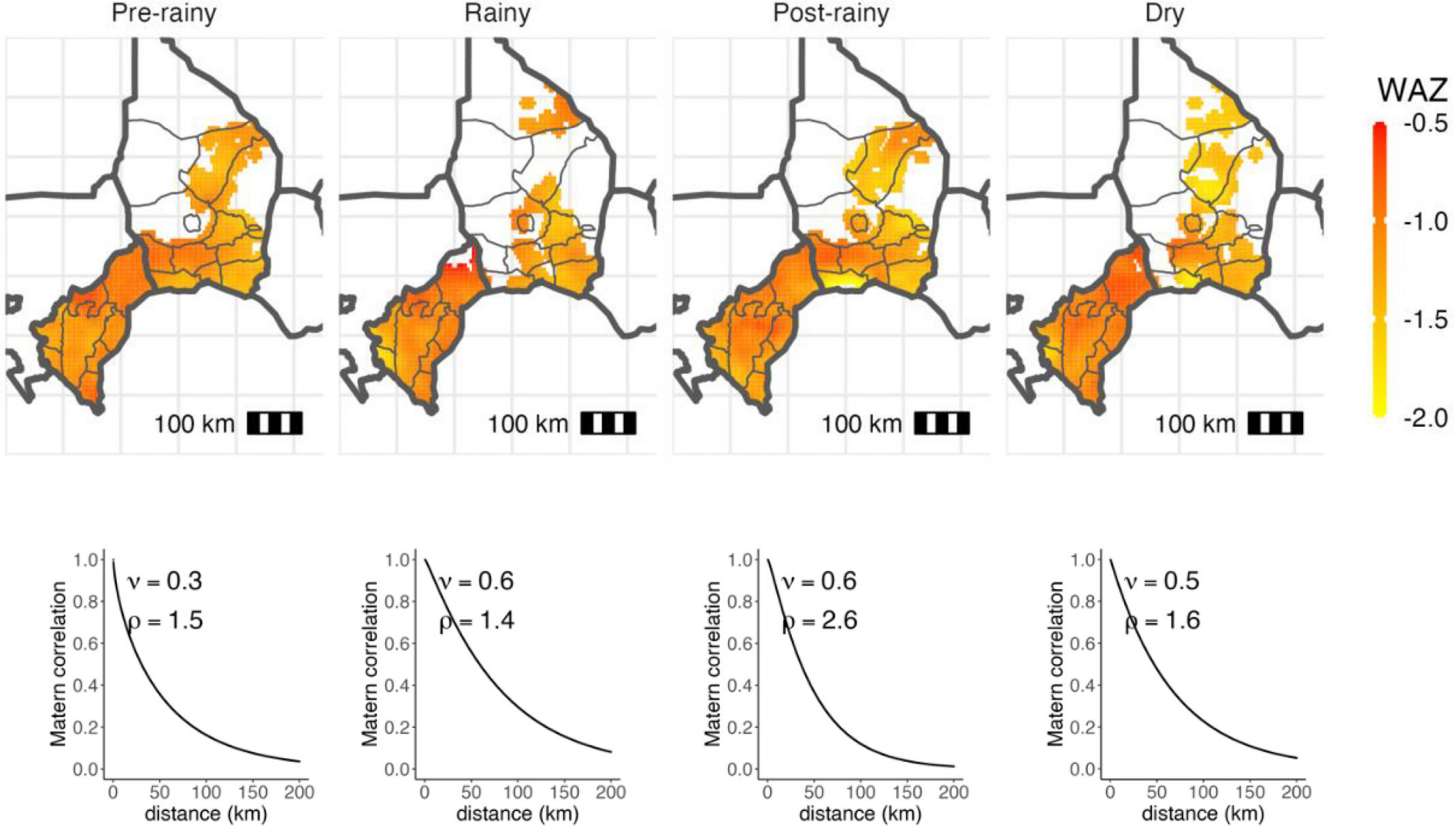
Spatial heterogeneity of weight-for-age z-score by season, visualised through simple kriging with a Matérn spatial correlation structure.

The sensitivity analysis by categorical season showed the lowest WAZ in the postrainy season (October to December) compared with the dry season of January to March (mean difference=−0.07; 95% CI −0.11 to −0.03; *P* value 0.002) (online supplemental table 1). Results were similar when looking at the prevalence of underweight with the postrainy season having a 2.1% (95%CI 0.9% to 3.3%) increase in prevalence as compared with the dry season (online supplemental table 2). As shown in figure 2, the primary nadir aligns with the highest temperatures, and the secondary nadir follows the season of most rainfall. Sensitivity analyses by subgroup produced similar results, with the analyses by year showing timing similar peaks and nadirs that align with temperature and rainfall patterns (online supplemental figure 1). The analysis by age group similarly identified the same seasonal patterns shown in the primary analysis, though the 6–11-month age group had lower overall WAZ across all seasons (online supplemental figure 2). Both age groups have a significant 6-month seasonal cycle; however, the 1–5-month-old group has a stronger pattern (sin4π of day=0.31 (95%CI 0.24 to 0.39), *p* value<0.001 vs 0.08 (95%CI 0.03 to 0.14), *p* value 0.004) (online supplemental table 1). The analysis by treatment received again showed similar seasonal patterns and did not vary by arm (online supplemental table 3 and figure 3). There was high spatial correlation of WAZ in each season with a Matérn correlation of approximately 0.6 up to 50 km, with the lowest WAZ in the more southern Dosso region compared with the Tahoua region (figure 3).

## Discussion

This analysis leveraged data collected from the AVENIR trial to assess the seasonality and spatial distribution of underweight among infants 1–11 months of age in rural areas of Niger. This dataset enabled us to address important gaps in the existing literature by focusing on underweight among infants under 1 year old. We found strong seasonal and spatial patterns of underweight in infants in this setting, with two clear seasonal peaks aligning with temperature and rainfall patterns, and lower seasonal WAZ in the southern communities in the study.

The use of the harmonic regression allowed us to use data at the day level and thus avoid the loss of precision and potential biased conclusions that can accompany aggregation.^8 13^ Also, the inclusion of two harmonic trends allowed us to discover two peaks and nadirs. With this approach, our results were similar to a study of the seasonality of wasting in African drylands. When looking at areas that are arid and follow traditional agriculture seasonal cycles, as is the case in Niger, this study found a primary peak of wasting in May to June, similar to our finding of a primary nadir of WAZ in early June.^12^ Some studies reported different findings; however, these studies analysed season as either two or four seasons of the year, and thus some precision may have been lost. One of these studies reported that wasting was highest in the rainy season, and another reported that wasting was highest in the dry season.^10 11^ While these differences may be due in part to important climate and geographic differences, the use of categorical season variables may have also limited these prior analyses. Analysing only categorical representations of seasonality as in the sensitivity analysis by season here would have led to missing the primary nadir occurring in May as the prerainy season of April to June was not found to be significantly different than the dry season of January to March (online supplemental table 2).

Harvest timing in Niger likely also contributes to the seasonality of underweight status. Niger has a high prevalence of food insecurity, with 33.3% of the rural population being classified as ‘at risk’ for food insecurity, 13.2% as moderately food insecure and 2.5% as severely food insecure.^28^ In 2019, the Global Hunger Index ranked Niger 101 out of 117 countries based on prevalence of wasting, stunting, undernourishment and child mortality.^29^ The World Bank states that over 80% of the population of Niger depends on agriculture.^30^ In Burkina Faso, crop yield has been linked to child survival for communities that rely on subsistence farming, particularly during the age of 6 months to 1.9 years old.^31^ Having only one harvest per year and being dependent on agriculture could contribute to the seasonal patterns of underweight status we see in our analysis. In Niger, harvest begins in August and concludes in December. In our analysis, we see a steady reduction in WAZ during these months; however, after the conclusion of harvest, WAZ starts to increase again until the primary peak in late February. This could reflect the greater availability of food during these months after harvest.

Currently, WHO guidelines use other anthropometric measures including weight-for-length z-score (WLZ) and mid-upper arm circumference (MUAC), as the primary enrolment criterion for malnutrition programmes.^14^ The guidelines acknowledge the use of WAZ as a general indicator of undernutrition but are not used as a standalone criterion for the diagnosis or management of malnutrition. The present study was unable to include analyses using these other indicators as weight was the only anthropometric measure included. However, understanding the seasonality and spatiality of WAZ still adds valuable insight to the literature, especially in the context of child mortality. A recent systematic review of studies conducted in low- and middle-income countries found using WAZ to be a better predictor of mortality for infants under 6 months old than WLZ.^32^ Further, across 12 studies in Africa and Asia, severe underweight status as measured by WAZ better identified children who died than using WHZ or MUAC.^33^ While wasting, as measured by WLZ, is the primary enrolment criterion for malnutrition programmes, WAZ is still an important indicator regarding mortality at the community level. Since the primary goal of most malnutrition programmes is to prevent mortality, the most effective case definitions are those that prioritise identifying children at the highest risk of death.^34^

There have been limited studies that have explored the spatial distribution of WAZ and other indicators of malnutrition. One study in Uganda found spatial dependencies and a spatial pattern of malnutrition in both stunting, wasting and underweight.^35^ Our analysis confirms these findings with high spatial correlation of WAZ in rural areas of Niger. The lowest WAZ was found in the southern region of Dosso, which has a more pronounced rainy season than the more arid northern region of Tahoua. Understanding the spatial patterns of malnutrition and how they interact with seasonality could be used to target higher prevalence areas of WAZ.

Strengths of this study include year-round data collection on WAZ and age due to the rolling census. As data were collected as part of a standardised randomised controlled trial, the data team was experienced and well-trained. The inclusion of underweight status for infants less than 6 months old adds crucial information for this age group that is not well represented in the literature. The WHO recently emphasised the importance of including infants under 6 months at risk of poor growth and development, which is a newly added guideline as of 2023.^14^ One way the WHO defines poor growth and development is through low WAZ.^14^ Further, this analysis included seasonal patterns over 2 years which confirmed that the seasonal patterns were consistent across years and were not due to specific events that happened in either year. Limitations include the lack of individual-level longitudinal data collection, given the community-randomised design of the trial. This precluded us from looking at change in WAZ in individual infants over time as it relates to seasonality. Similarly, the large scale of the trial resulted in limited data collection overall, so we were unable to evaluate the impact of other factors on WAZ patterns over time, or other indicators of malnutrition. Further, the data collected was limited to only 2 years and two regions in Niger. Thus generalisability is limited to regions within West Africa which follow a similar seasonal pattern as the regions of Dosso and Tahoua in Niger.

Overall, we found that WAZ is lowest during the months of May and November, which correspond to the prerainy/high temperature and postrainy seasons in Niger. The strong seasonal pattern of underweight in rural Niger that we found here could be used for targeting the time programmes aiming to decrease the burden of underweight among infants in this setting.

## supplementary material

10.1136/bmjgh-2024-017643 online supplemental file 1

10.1136/bmjgh-2024-017643 online supplemental file 2

## Data Availability

Data are available in a public, open access repository.
