# Supplementary material for: Seasonality of underweight among infants 1–11 months old in Niger: an exploratory analysis of data from a cluster-randomised trial
Source: BMJ Glob Health. 2025 Mar 28;10(3):e017643. doi: 10.1136/bmjgh-2024-017643 (PMC11956403; doi:10.1136/bmjgh-2024-017643)

Supplemental Material

Supplemental Table 1. Mean difference in WAZ by season

Season	WAZ<br>Mean (SD)	Mean Difference<br>(95% CI)	P-value
Dry (January to March)	-1.1 (0.08)	Ref	Ref
Pre-rainy (April to June)	-1.2 (0.6)	-0.03 (-0.07 to 0.01)	0.176
Rainy (July to September)	-1.1 (0.7)	0.00 (-0.05 to 0.05)	0.979
Post-rainy (October to December)	-1.2 (0.7)	-0.07 (-0.11 to -0.03)	0.002

Supplemental Table 2. Mean difference in prevalence of underweight as defined by having WAZ < -2 by season

**Season**	**Mean Prevalence (SD)**	**Mean Difference (95% CI)**	**P-value**
Dry (January to March)	24.7%	Ref	Ref
Pre-rainy (April to June)	25.7%	1.0% (-0.2% to 2.2%)	0.103
Rainy (July to September)	25.8%	1.1% (-0.3% to 2.4%)	0.121
Post-rainy (October to December)	26.8%	2.1% (0.9% to 3.3%)	<0.001

Supplemental Table 3. Harmonic Regression Coefficients, 95% Confidence Intervals, and *P*-values for the seasonal trends of WAZ by subgroups

Group	Coefficient	Estimate (95% CI)	P-value
**Year**			
**2021**	Day	0.00 (0.00 to 0.00)	0.2158
	sin2π of day	-0.03 (-0.17 to 0.10)	0.6206
	cos2π of day	-0.03 (-0.10 to 0.04)	0.4219
	sin4π of day	0.17 (0.07 to 0.26)	0.0010
	cos4π of day	0.03 (-0.04 to 0.10)	0.4144
	Constant	-0.91 (-1.11 to -0.72)	< 0.001
**2022**	Day	0.00 (0.00 to 0.00)	0.0198
	sin2π of day	0.08 (-0.02 to 0.17)	0.1160
	cos2π of day	-0.02 (-0.07 to 0.04)	0.4850
	sin4π of day	0.10 (0.03 to 0.18)	0.0091
	cos4π of day	-0.07 (-0.12 to -0.01)	0.0016
	Constant	-1.36 (-1.49 to -1.23)	<0.001
**Age Group**			
**1-5m**	Day	0.00 (0.00 to 0.00)	0.2233
	sin2π of day	0.06 (-0.05 to 0.16)	0.2857
	cos2π of day	0.02 (-0.03 to -0.08)	0.4528
	sin4π of day	0.31 (0.24 to 0.39)	<0.001
	cos4π of day	-0.01 (-0.06 to 0.04)	0.6959
	Constant	-0.71 (-0.85 to -0.57)	<0.001
**6-11m**	Day	0.00 (0.00 to 0.00)	0.0049
	sin2π of day	0.11 (0.03 to 0.18)	0.0065
	cos2π of day	0.06 (0.02 to 0.11)	0.0025
	sin4π of day	0.08 (0.03 to 0.14)	0.0035
	cos4π of day	0.01 (-0.03 to 0.05)	0.7127
	Constant	-1.52 (-1.62 to -1.42)	<0.001
**Treatment Received**			
**Azithromycin**	Day	0.00 (0.00 to 0.00)	<0.001
	sin2π of day	0.15 (0.05 to 0.24)	0.002
	cos2π of day	-0.01 (-0.06 to 0.04)	0.6596
	sin4π of day	0.23 (0.17 to 0.30)	<0.001
	cos4π of day	-0.01 (-0.07 to 0.04)	0.5799
	Constant	-1.32 (-1.46 to -1.19)	<0.001
**Placebo**	Day	0.00 (0.00 to 0.00)	<0.001
	sin2π of day	-0.03 (-0.13 to 0.08)	0.5873
	cos2π of day	-0.05 (-0.11 to 0.01)	0.0867
	sin4π of day	0.10 (0.02 to 0.17)	0.0106
	cos4π of day	-0.01 (-0.07 to 0.04)	0.7140
	Constant	-1.10 (-1.24 to -0.95)	<0.001

Supplemental Figure 1. Predicted Daily Values of WAZ by Year from Harmonic Regression Model

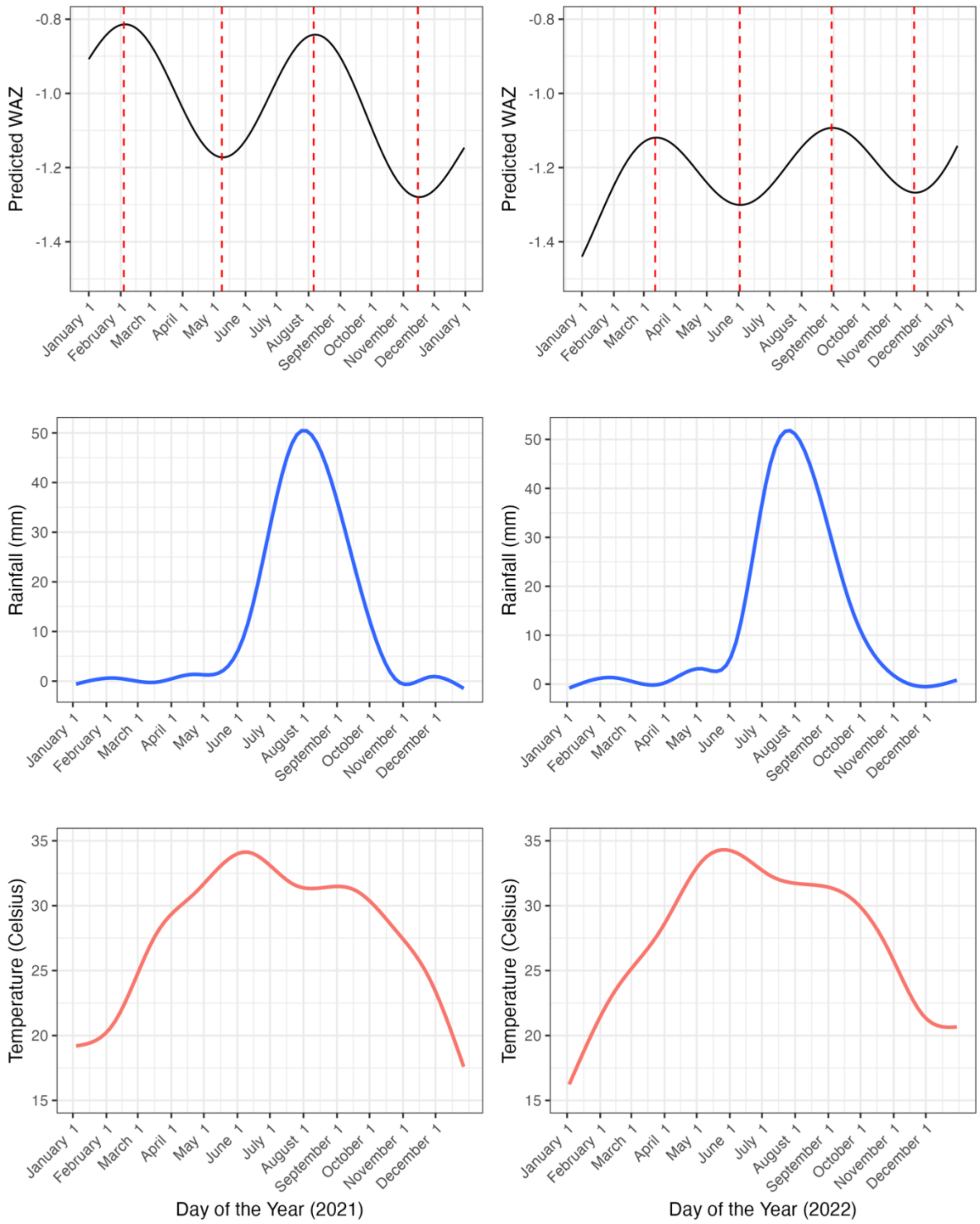

Supplemental Figure 2. Predicted Daily Values of WAZ by Age Group from Harmonic Regression Model

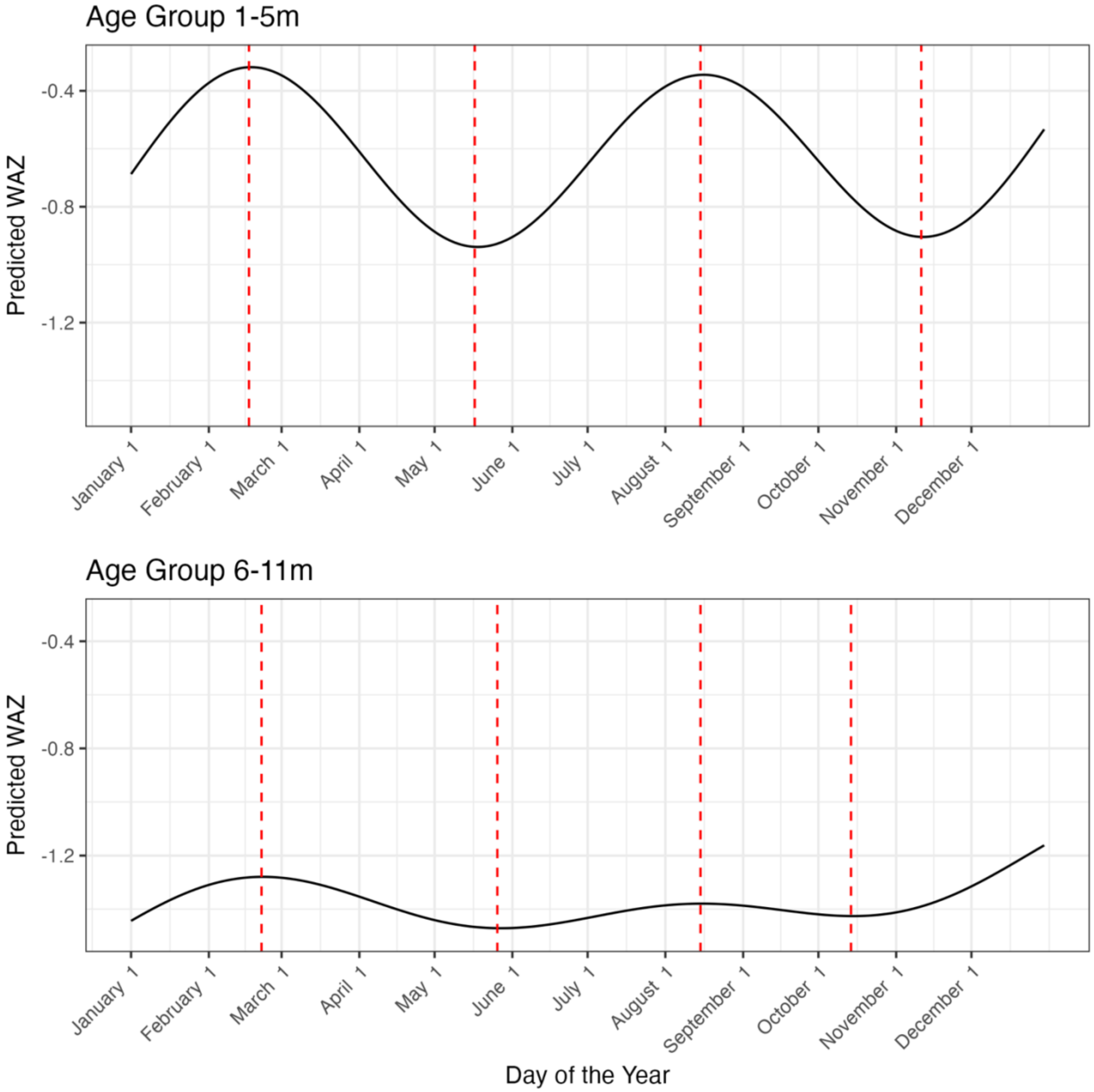

Supplemental Figure 3. Predicted daily values of WAZ by treatment received from harmonic regression model

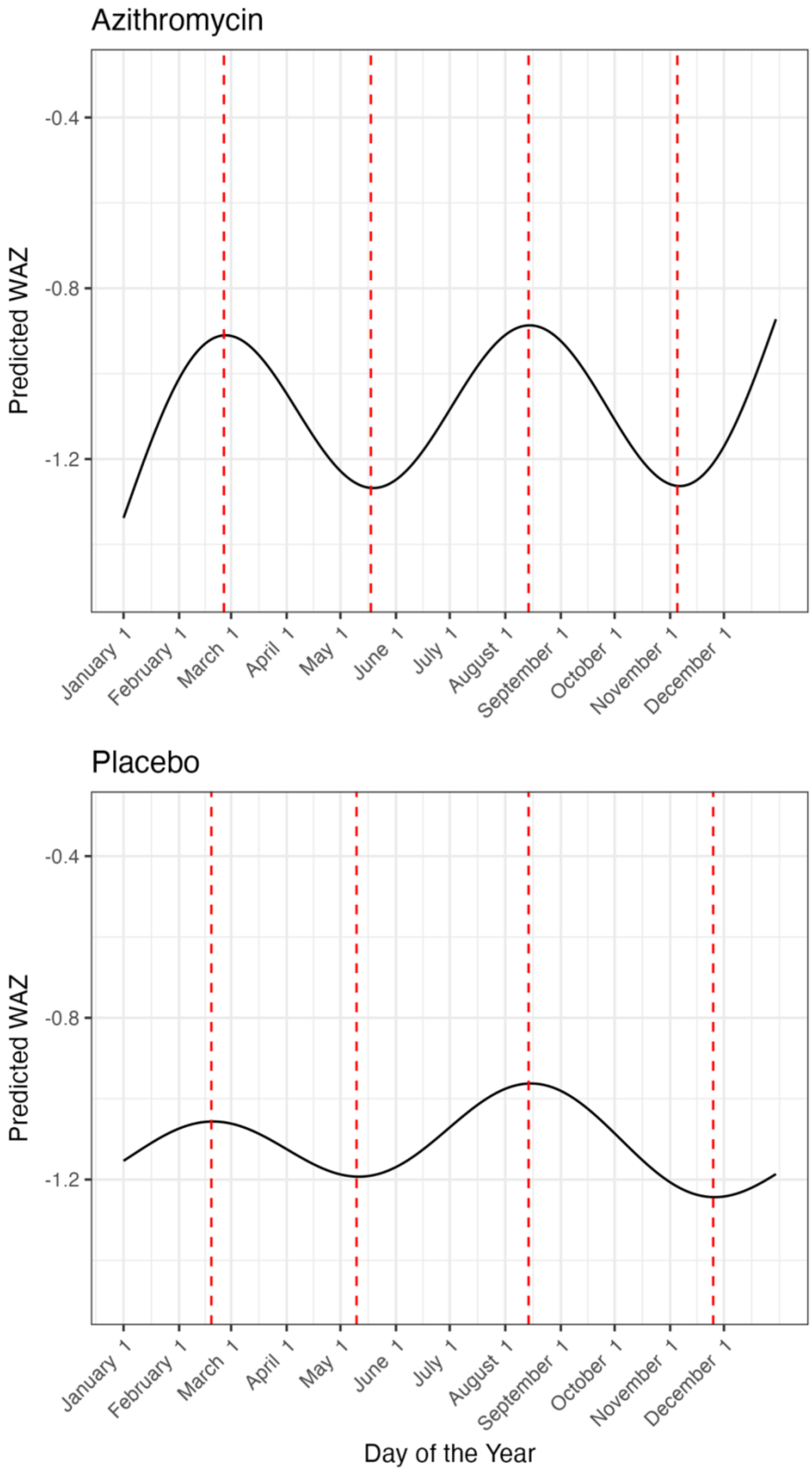

Supplement: online supplemental file 2 [file bmjgh-10-3-s002.pdf]
